# Ependymal and Neural Stem Cells of Adult Molly Fish (*Poecilia sphenops*, Valenciennes, 1846) Brain: Histomorphometry, Immunohistochemical, and Ultrastructural Studies

**DOI:** 10.3390/cells11172659

**Published:** 2022-08-27

**Authors:** Doaa M. Mokhtar, Ramy K. A. Sayed, Giacomo Zaccone, Marco Albano, Manal T. Hussein

**Affiliations:** 1 Department of Cell and Tissues, Faculty of Veterinary Medicine, Assuit University, Assiut 71526, Egypt; 2 Department of Anatomy and Embryology, Faculty of Veterinary Medicine, Sohag University, Sohag 82524, Egypt; 3 Department of Veterinary Sciences, Polo Universitario dell’Annunziata, University of Messina, 98168 Messina, Italy; 4 Department of Chemical, Biological, Pharmaceutical and Environmental Sciences, University of Messina, 98166 Messina, Italy

**Keywords:** ependymal cells, stem cells, neurogenesis, teleosts, glial cells, Sox9, GFAP

## Abstract

This study was conducted on 16 adult specimens of molly fish (*Poecilia sphenops*) to investigate ependymal cells (ECs) and their role in neurogenesis using ultrastructural examination and immunohistochemistry. The ECs lined the ventral and lateral surfaces of the optic ventricle and their processes extended through the tectal laminae and ended at the surface of the tectum as a subpial end-foot. Two cell types of ECs were identified: cuboidal non-ciliated (5.68 ± 0.84/100 μm^2^) and columnar ciliated (EC3.22 ± 0.71/100 μm^2^). Immunohistochemical analysis revealed two types of GFAP immunoreactive cells: ECs and astrocytes. The ECs showed the expression of IL-1β, APG5, and Nfr2. Moreover, ECs showed immunostaining for myostatin, S100, and SOX9 in their cytoplasmic processes. The proliferative activity of the neighboring stem cells was also distinct. The most interesting finding in this study was the glia–neuron interaction, where the processes of ECs met the progenitor neuronal cells in the ependymal area of the ventricular wall. These cells showed bundles of intermediate filaments in their processes and basal poles and were connected by desmosomes, followed by gap junctions. Many membrane-bounded vesicles could be demonstrated on the surface of the ciliated ECs that contained neurosecretion. The abluminal and lateral cell surfaces of ECs showed pinocytotic activities with many coated vesicles, while their apical cytoplasm contained centrioles. The occurrence of stem cells in close position to the ECs, and the presence of bundles of generating axons in direct contact with these stem cells indicate the role of ECs in neurogenesis. The TEM results revealed the presence of neural stem cells in a close position to the ECs, in addition to the presence of bundles of generating axons in direct contact with these stem cells. The present study indicates the role of ECs in neurogenesis.

## 1. Introduction

*Poecilia sphenops* (Valenciennes, 1846) is a freshwater fish species commonly named molly fish. They are natural inhabitants in the freshwater streams and coastal brackish water of Mexico and Colombia, and can be found in many countries around the world through the aquarium fish trade [1]. They are mostly observed in swarms below floating vegetation as they feed principally on algae and other herbal resources [2]. Because of their higher birth size, growth rate, reproduction, and brood number, mollies are categorized as one of the most popular fish. The lifespan of the fish is 3–5 years, and they reach maturity at the age of 4 months. Moreover, this species is a member of the viviparous fish [3,4]. In mammals, more than 60–75% of the cerebrospinal fluid (CSF) is mainly secreted by the four choroid plexuses (CP), one in each ventricle. The remaining 20–30% is produced by the ventricular ependymal cells and the blood–brain barrier (BBB) [5]. From the lateral ventricles, where CSF is produced, it flows unidirectionally in a craniocaudal direction into the arachnoid granulations, where it is reabsorbed into the dural venous sinuses. A pump-like activity forces the liquid through the ventricular system and this action is accommodated by the rhythmic beating of the ependymal cilia [6]. A large range of mammalian homeostatic activities, including the transportation of nutrients, the elimination of catabolic waste products, the absorption of hydro-mechanical stress, the control of thermal stress, and the transfer of neurotransmitters, are carried out by CSF [7]. In zebrafish juveniles and adults, the circulation of CSF has recently been reported to be essential for the body axis formation, in addition to embryo and spine morphogenesis [8,9].

Ependymal cells (ECs) are glial cells that line the ventricular system of the brain and the central canal of the spinal cord, forming an epithelial barrier called the ependyma. ECs play an important role in the process of neurogenesis, neuronal differentiation, and axonal guidance [10]. ECs are multi-ciliated cells, and the motility of cilia generates the directional flow and homeostasis of the cerebrospinal fluid (CSF) inside the brain ventricles and spinal cord [11,12]. The motility of cilia in zebrafish regulates the morphogenesis of the spine through CSF circulation and the formation of a glycoprotein filament called Reissner fiber [9,13]. Furthermore, ependymal cells act as a moderator between the parenchyma and cerebrospinal fluid-filled cavities throughout life. Therefore, these cells regulate the bidirectional flow of immune cells and solutes between the CSF and interstitial fluid [14,15]. In addition to the role of ECs in CSF circulation, they are involved in the adult neurogenic niche that assembles into a characteristic pinwheel-like organization [16]. The neurogenic niche is restricted in the mammalian brain to certain regions such as the olfactory bulb, the subventricular zone (SVZ) of the lateral ventricles and the dentate gyrus (DG) of the hippocampus [17]. In contrast to the brain of mammals, the adult teleost fish has several sites containing neural stem cells (NSCs) throughout the rostrocaudal axis of the brain [18,19]. The optic tectum of zebrafish is one of the neurogenic niches that contains several proliferating neural stem cells [20].

ECs were studied at the ultrastructural level in the bluegill *Lepomis macrochirus*, the barred sand bass *Palarabrax nebulifer* [21], and in the optic tectum of goldfish *Carassius auratus* [22]. The morphology of the central nervous system shows distinct variations among fish species [23]. In fish, ECs comprise the main neurological element in the brain, as they are fundamental for CSF circulation, in addition to their roles in the adult neurogenic niche [15,24]. The neurogenic niche of the adult telencephalic ventricle of zebrafish is composed of multi-ciliated ependymal cells and radial glial cells, which act as neural stem cells and generate neuronal progenitor cells and migrating new neurons [18]. Little data are available regarding the general structure of fish ependymal cells. Therefore, the present work aims to investigate the morphology of the ependymal cells lining the surface of the optic tectum of molly fish and their role in neurogenesis using ultrastructural examination and different immunostaining. Obtaining this region for our study was very easy, as ependymal cells line the ventricular system of the brain, and the optic tectum is the most obvious part of the mesencephalon.

## 2. Materials and Methods

The present study was conducted according to the University guidelines and Egyptian legal requirements for animal care. All the procedures of this work were approved by the National Ethical Committee of the Faculty of Veterinary Medicine at Assiut University in Egypt.

### 2.1. Sample Collection

This study was performed on randomly selected adult male molly fish (*Poecilia sphenops*, *n* = 16, with age ranging from 9 to 10 months), which were bought from an ornamental fish shop. The anthropometric characteristics of the selected specimens were 4.20 ± 4.0 cm standard length and 10.60 ± 1.70 gm body weight.

### 2.2. Semithin Sections and Transmission Electron Microscopy (TEM)

Small brain samples were left overnight in a mixture of paraformaldehyde–glutaraldehyde for proper fixation [25,26]. The specimens were then washed using 0.1 mol/L phosphate buffer and were osmicated using 1% osmium tetroxide. Following that, the samples were passed in ethanol for dehydration, and were transferred to propylene oxide. Finally, the tissues were embedded in Araldite. Semithin sections of about 1 μm thick were cut and were stained with toluidine blue for light microscopy examination; meanwhile, ultrathin sections of about 70 nm were cut by Ultrotom-VRV (LKB, Bromma, Germany) and were examined using a JEOL-100CX II electron microscope (Boston, MA, USA) after staining with lead citrate and uranyl acetate [27].

### 2.3. Immunohistochemical Analysis

The Pierce Peroxidase Detection Kit (36000, Thermo Fisher Scientific, Waltham, MA, USA) was used for the immunohistochemical analysis of brain sections. Firstly, the sections were embedded in xylene for deparaffinization, passed in graded series of ethanol for rehydration, and finally were washed using distilled water [28]. Following washing, the epitope exposure was increased through heating sections in a 0.01 M sodium citrate buffer (pH 6.0) using a microwave for 15 min. The tissues were kept for 30 min at room temperature for cooling, and then were washed with wash buffer consisting of 0.05% Tween-20-contained tris-buffered saline. To knock endogenous peroxidase activity out, the sections were incubated in peroxidase suppressor for 30 min. Following that, tissues were washed using the wash buffer and were blocked for 30 with universal blocker™ blocking buffer at room temperature. After blocking, the sections underwent overnight incubation at 4 °C with primary antibodies (dilution 1:100) against interleukin 1 beta (IL-1β) (sc-7884, Santa Cruz Biotechnology, Heidelberg Germany), glial fibrillary acidic protein (GFAP) (PA5-16291, Thermo Fisher Scientific, Waltham, MA, USA), autophagy protein 5 (APG5) (sc-133158, Santa Cruz Biotechnology, Heidelberg, Germany), nuclear factor erythroid 2-related factor 2 (Nrf2) (sc-722, Santa Cruz Biotechnology, Heidelberg, Germany), myostatin (AB3239, Sigma-Aldrich, Madrid, Spain), SRY-Box transcription factor 9 (Sox9) (AB5535, Sigma-Aldrich, Madrid, Spain), CD3 (ab828, Abcam, Cambridge, UK), and S100 protein (Z0311, Dako, Glostrup, Denmark). In parallel, tissue specimens, in which S100 protein primary antibody was omitted and replaced with buffer, served as negative controls. The tissues were washed using wash buffer and were then incubated at room temperature for 30 min with diluted goat anti-mouse IgG (31800, Invitrogen, Waltham, MA, USA, dilution 1:100) and diluted goat anti-rabbit IgG (65-6140, Invitrogen, Waltham, MA, USA, dilution 1:1000) secondary antibodies. After incubation, the sections were washed with a wash buffer, and were incubated for 30 min with Avidin-HRP (43-4423, Invitrogen, Waltham, MA, USA, dilution the diluted 1:500) in the blocking buffer. The tissues were washed with a wash buffer and were incubated for 5–15 min with a 1× metal-enhanced DAB substrate working solution. After obtaining the desired staining, the sections were finally washed with the wash buffer, counterstained with Harris modified hematoxylin stain, and mounted with mounting media provided in the detection kit [29,30].

### 2.4. Morphometrical and Quantitative Studies

Morphometrical and quantitative measurements were carried out on brain images of semithin sections and immunohistochemical-stained sections, respectively, using Image-J software [31,32]. The number of ependymal cells per constant area of 100 μm^2^ was measured. The expression intensity (EI) of the immunohistochemistry staining markers was quantified using ImageJ software (1.48v), and a color deconvolution algorithm was used to differentiate and isolate different stains for their quantification. The optical density of the red, blue and green color vectors was calculated for each one using default software settings and control slides stained separately with hematoxylin or DAB [33,34]. All these measurements were performed on a total number of 10 brains, and five randomly selected sections per fish were examined (from each section, three randomly selected regions were measured). The obtained morphometric data are presented as mean ± SEM.

### 2.5. Digitally Colored TEM Images

Ependymal cells, neurons, and other specific cellular elements were digitally colored using Adobe Photoshop software (version 6) to boost the visual dissimilarity between the abundant structures depicted in single electron micrograph.

## 3. Results

### 3.1. Histological Analysis

**Figure 1:**
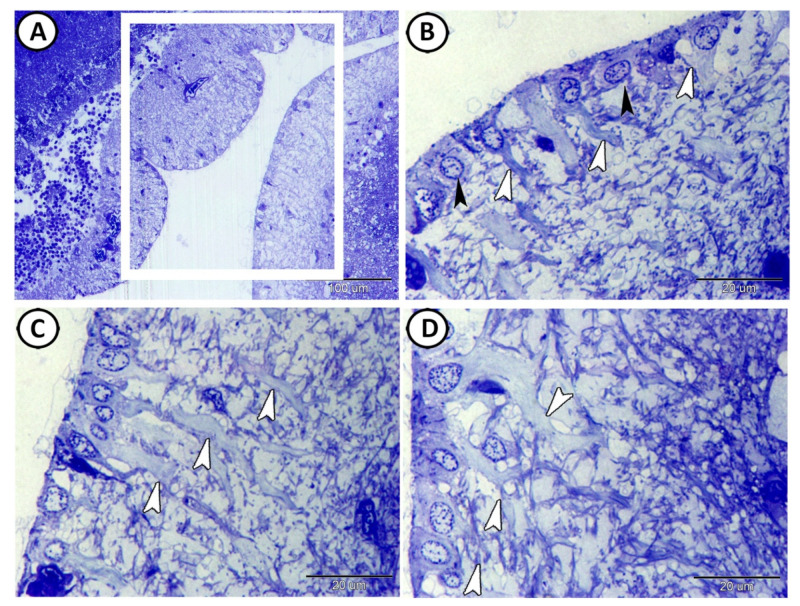
Semithin sections of the ependymal cells (ECs) were stained with toluidine blue. (**A**) ECs lined the surfaces of the optic ventricle (boxed area). (**B**) Two cell types of EC based on shape were identified: cuboidal non-ciliated (black arrowheads) and columnar ciliated ECs (white arrowheads). (**C**,**D**) The basal processes of the columnar ECs ramified within the ependymal region to form the characteristic fibrous meshwork (white arrowheads).

The ependymal cells (EC) lined the ventral and lateral surfaces of the optic ventricle (Figure 1A). Two cell types of EC based on shape were identified: cuboidal non-ciliated and columnar ciliated EC (Figure 1B). Morphometric analysis of the brain sections of the molly fish revealed that the number of ciliated ECs/100 μm^2^ was 3.22 ± 0.71, while the number of non-ciliated ECs was 5.68 ± 0.84. The columnar cells showed many basal processes that ramified and interweaved within the ependymal region to form the characteristic fibrous meshwork (Figure 1C,D).

**Figure 2:**
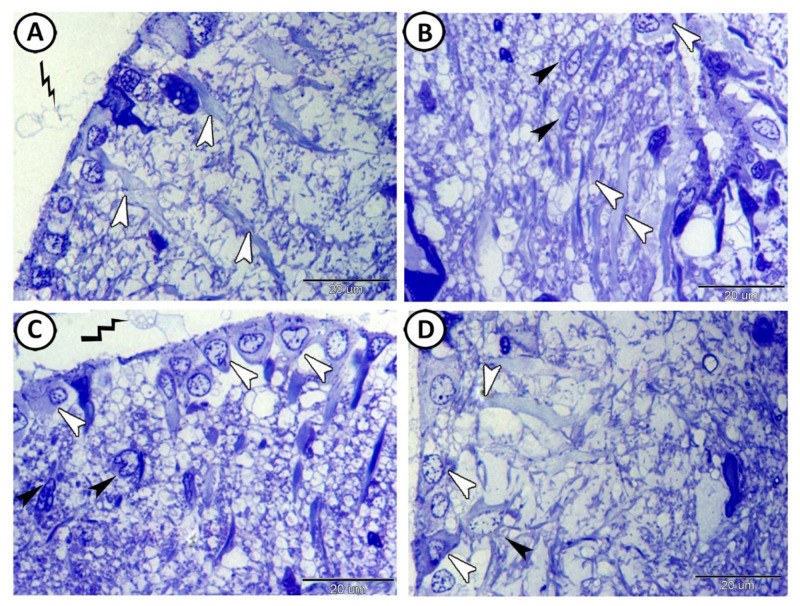
Semithin sections of the ependymal cells (ECs) were stained with toluidine blue. (**A**) The processes of ECs (white arrowheads) extended through the full thickness of the tectal laminae. Note the apical neurosecretion (zigzag black line). (**B**,**C**) The basal processes of the EC (white arrowheads) were connected with stem cells (black arrowheads). Note that the surfaces of the ECs were covered with secretion (zigzag black line). (**D**) The interaction between the ECs (white arrowheads) and multipolar neurons (black arrowheads).

The processes of ECs extended through the full thickness of the tectal laminae and ended at the surface of the tectum as a subpial end-foot (Figure 2A), while the surface of these cells was covered with secretion (Figure 2A,C). Intermingling with the basal processes of the ECs were cell bodies of stem cells that were characterized by a high nuclear cytoplasmic ratio and divided nuclei (Figure 2B,C). In addition, multipolar neurons were observed in contact with the processes of these ECs (Figure 2D).

### 3.2. Immunohistochemistry

**Figure 3:**
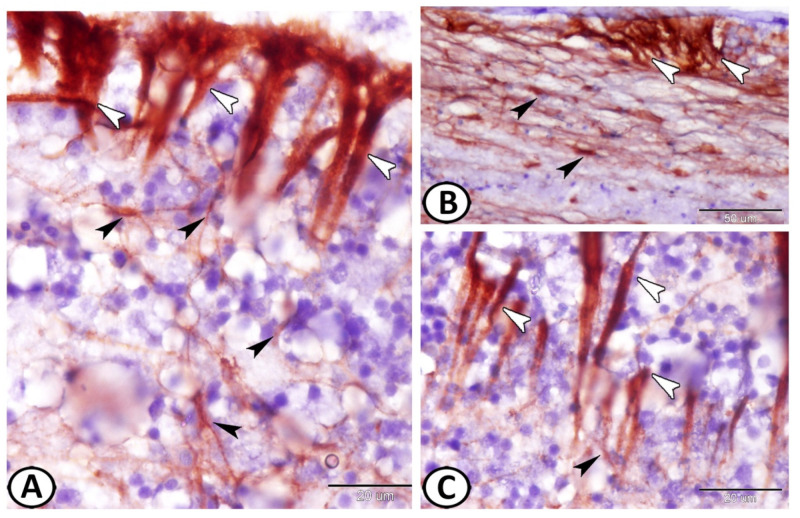
GFAP immunohistochemistry. (**A**–**C**) Two types of GFAP immunoreactive cells could be identified: ependymal cells (white arrowheads) and astrocytes (black arrowheads). Note the intense reactions of ECs and the processes of astrocytes that were in direct contact with the processes of the ECs.

Immunohistochemical analysis revealed the presence of two types of GFAP immunoreactive cells with EI of 20.44 ± 1.7%: ependymal cells (EC) and astrocytes (Figure 3A). The ECs showed more intense reactions than those of the astrocytes. The processes of the astrocytes were in direct contact with the processes of the ECs (Figure 3A–C).

**Figure 4:**
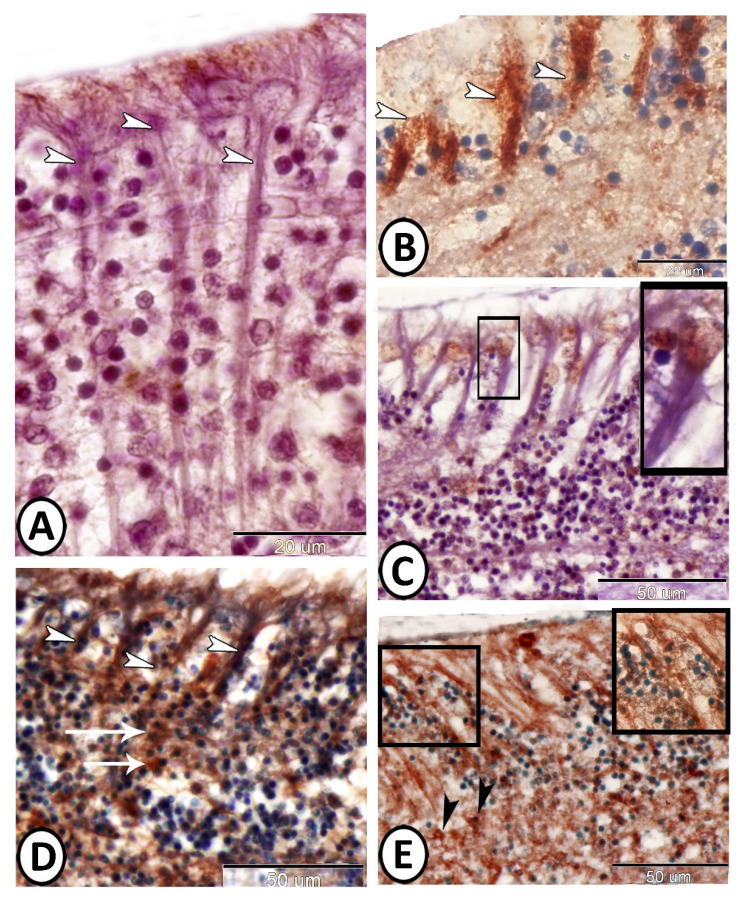
Immunohistochemistry of ECs. (**A**) ECs showed expression of IL-1β (white arrowheads) in their cytoplasmic processes. (**B**) ECs showed strong cytoplasmic expression of APG-5 (white arrowheads). (**C**) ECs showed nuclear expression of Nfr2 (boxed areas). (**D**,**E**) ECs showed expression of myostatin (white arrowheads) and SOX-9 (boxed areas), respectively, in their cytoplasmic processes. Note the proliferative activity of the neighboring stem cells (black arrowheads and white arrows).

The ECs showed an expression of IL-1β (Figure 4A) in their cytoplasmic processes with an EI of 33.12 ± 2.68%. ECs also showed strong cytoplasmic expressions of APG5, where the EI was 15.41 ± 1.49% (Figure 4B). On the other hand, ECs showed nuclear expressions of Nfr2, with an EI of 17.47 ± 1.78% (Figure 4C). Moreover, ECs showed expressions of myostatin (EI was 33.9 ± 2.65%, Figure 4D) and SOX9 (EI was 46.74 ± 3.05%, Figure 4E) in their cytoplasmic processes. The proliferative activity of the neighboring stem cells was also distinct (Figure 4D,E).

**Figure 5:**
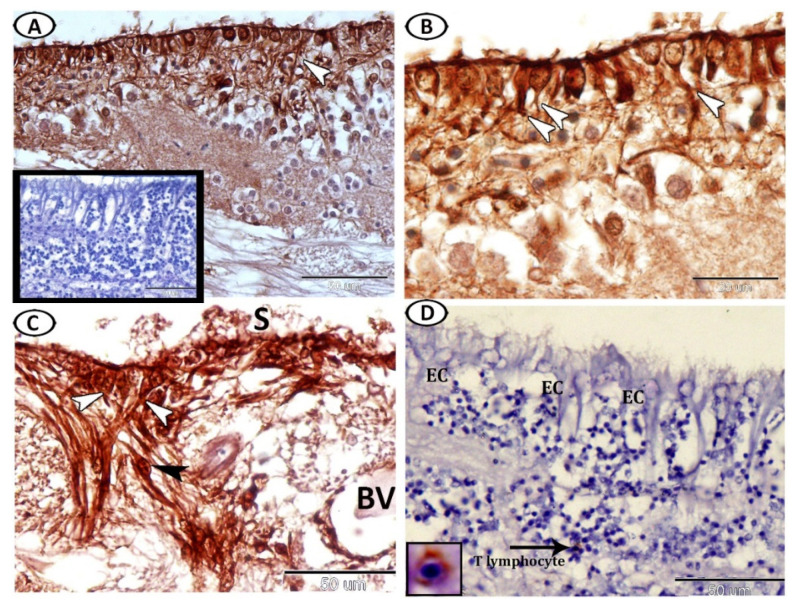
Immunohistochemistry of S100 protein and CD3 expression in the wall of the adult mesencephalic ventricle of molly fish. (**A**,**B**) The ependymal cells and their cytoplasmic processes (arrowheads) express S100 protein. The boxed area is a negative control. (**C**) Neurogenic niche showing immunostaining for S100 protein in the astrocytes (black arrowhead) and the ependymal cells and their long processes (white arrowheads). Note the blood vessel (BV) and secretion (S). (**D**) T lymphocytes in the neurogenic niche express CD3 (arrow, boxed area), while ependymal cells (EC) are negatively stained to CD3.

The expression of S100 protein in the wall of the adult mesencephalic ventricle was investigated. We observed S-100 protein expression in the cytoplasm and the nucleus of ECs as well as in their cytoplasmic processes. However, the neural stem cells showed negative immunoreactivity for S100 protein (Figure 5A,B). Moreover, the sub-ependymal astrocytes expressed S100 protein (Figure 5C). CD3 showed singly distributed T lymphocytes in the subependymal region and negative-stained ependymal cells (Figure 5D). The typical morphology of T cells includes a small, round, and dark nucleus with a scarce cytoplasm.

### 3.3. Transmission Electron Microscopy (TEM)

**Figure 6:**
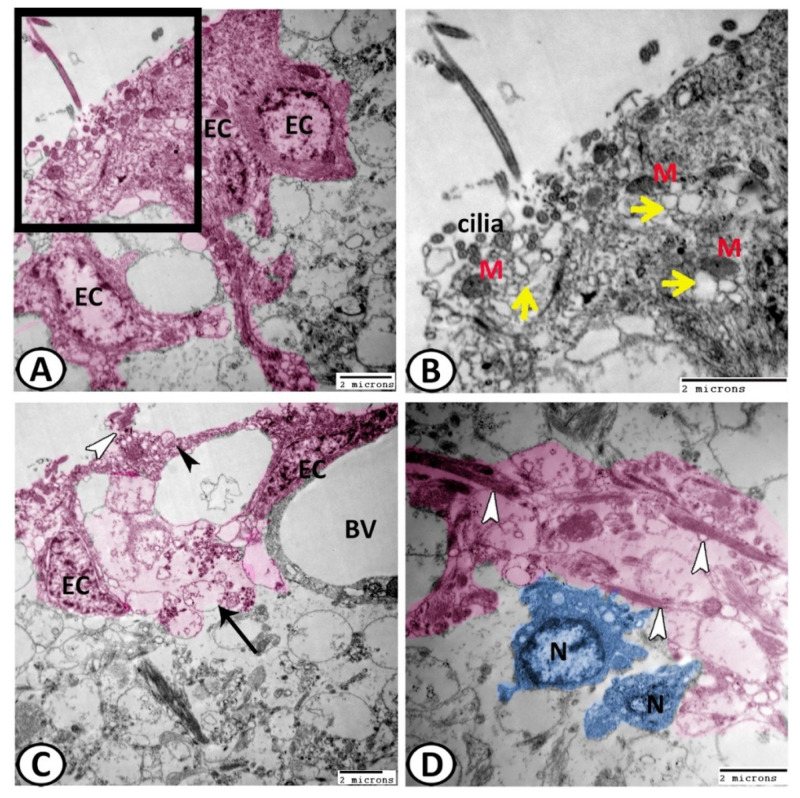
Digital colored transmission electron microscopy (TEM) of EC (pink). (**A**,**B**) The ciliated ECs were covered by cilia and their apical cytoplasm showed many mitochondria (M) and many vesicles of different shapes (yellow arrowheads). (**C**) The non-ciliated ECs showed bleb-like protrusions (white arrowhead) containing clear vesicles (black arrowhead). Some non-ciliated ECs were branched to encircle the nearby blood vessels (BV). The ECs gave off collateral projections that were associated with the synapse (black arrow). (**D**) Distinct glia–neuron interactions were observed between the processes of ECs (white arrowheads) with the progenitor neuronal cells (N, blue).

TEM revealed the presence of two types of cells: ciliated and non-ciliated cells. The ciliated cells possessed a euchromatic nucleus and were covered by cilia and their cytoplasm showed numerous mitochondria and many vesicles of different shapes (Figure 6A,B). However, the non-ciliated cells appeared smaller in size with fewer organelles and in many locations showed bleb-like protrusions containing clear vesicles (Figure 6C). Some non-ciliated ECs were branched to encircle the nearby blood vessels. The ECs gave off collateral projections that were associated with the synapse (Figure 6C). The most interesting finding in this study was the glia–neuron interaction that was observed (Figure 6D), where the processes of ECs met the progenitor neuronal cells in the ependymal area of the ventricular wall.

**Figure 7:**
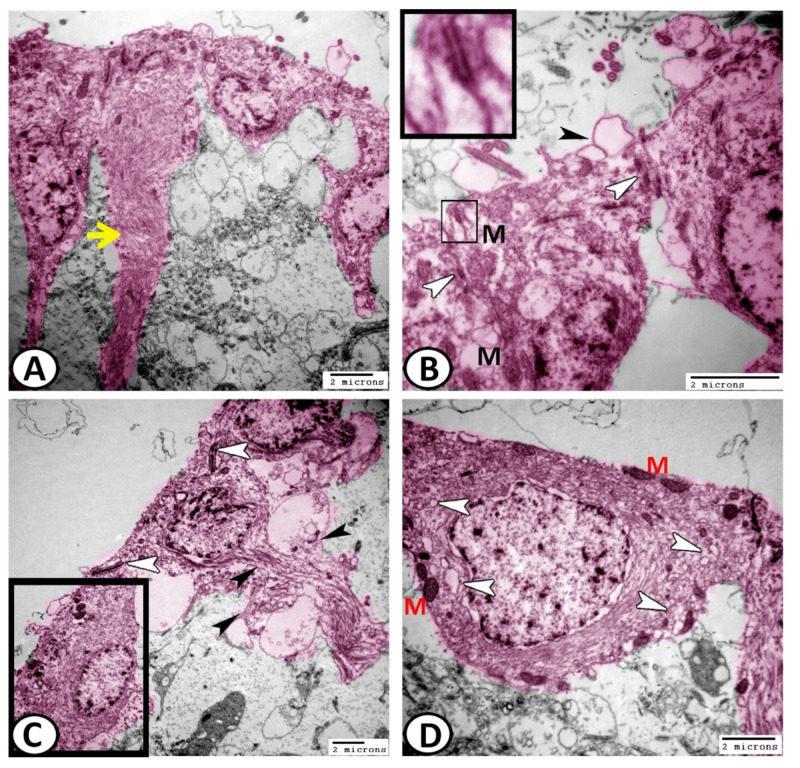
Digital colored transmission electron microscopy (TEM) of ECs (pink). (**A**) The ECs showed bundles of intermediate filaments (yellow arrowhead) in their processes and basal poles. (**B**) The ECs were connected by desmosomes (boxed areas) and gap junctions (white arrowheads). Note the presence of surface membrane-bounded vesicles (black arrowhead) containing neurosecretion and mitochondria (M). (**C**) The ECs showed distinct fascicle filaments that contained intermediate filaments (black arrowheads). Note the junctional complexes between the cells (white arrowheads). (**D**) Higher magnification of the boxed area of (**C**) shows the non-ciliated ECs that possessed many mitochondria (M) and abundant vesicles (white arrowheads).

These cells also showed bundles of intermediate filaments in their processes and basal poles (Figure 7A,C). The ECs were connected by desmosomes, followed by gap junctions (Figure 7B). Many membrane-bounded vesicles could be demonstrated on the surface of the ciliated ECs that contained neurosecretion (Figure 7B). The ciliated cells also contained distinct fascicles of intermediate filaments (Figure 7C). However, the non-ciliated ECs possessed many mitochondria and abundant vesicles, which were distributed all over the cytoplasm (Figure 7D).

**Figure 8:**
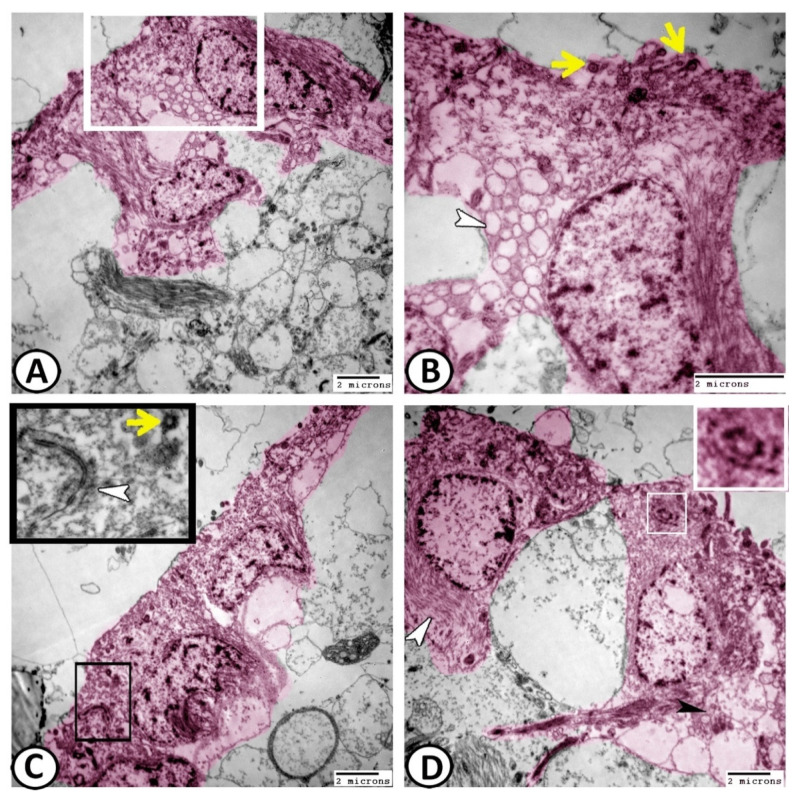
Digital colored transmission electron microscopy (TEM) of ECs (pink). (**A**,**B**) The surfaces of ciliated ECs showed many cross-sections of cilia (yellow arrowheads). The abluminal and lateral cell surfaces of ECs showed pinocytotic activities with many coated vesicles (white arrowhead). (**C**) The apical cytoplasm of ECs possessed centriole (yellow arrowhead) and well-developed junctional complexes (white arrowhead in the boxed area). (**D**) The cytoplasm of these ECs also contained bundles of intermediate filaments (white arrowhead), distinct fascicle filaments (black arrowhead) and centrioles (boxed areas).

EC’s abluminal and lateral cell surfaces showed pinocytotic activities with many coated vesicles (Figure 8A,B). The apical cytoplasm of the ECs contained centrioles (Figure 8C,D). The cytoplasm of these ECs also contained distinct fascicle filaments that contained intermediate filaments and mitochondria (Figure 8A,D).

**Figure 9:**
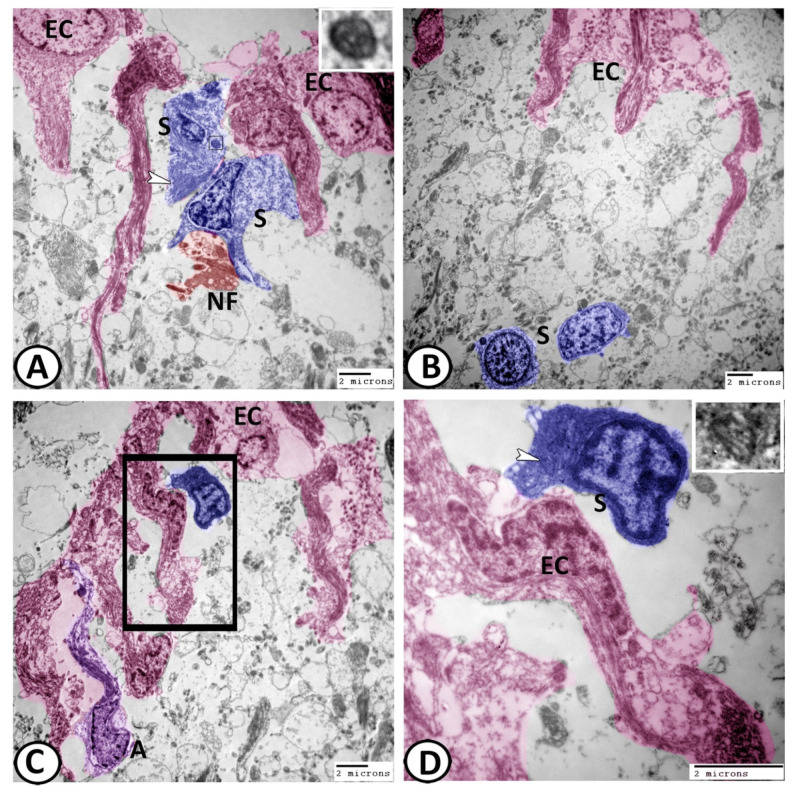
Digital colored transmission electron microscopy (TEM) of ECs (pink), astrocytes (violet), and stem cells (blue). (**A**,**B**) Many stem cells (blue, S) could be demonstrated among the ECs. In (**A**), bundles of intermediate filaments (white arrowhead) and centrioles (boxed areas) were observed in the cytoplasm of stem cells, while generated axons (NF, red) were observed in direct contact with these stem cells. (**C**) The processes of astrocytes (A, violet) and stem cells (blue) were seen among the ECs. (**D**) In addition, the stem cell cytoplasm contained centrioles (boxed area, white arrowheads).

Many stem cells with a characteristic high nuclear-to-cytoplasmic ratio and a rich cytoplasm with ribosomes could be demonstrated among the ECs (Figure 9A–D). Centrioles were considered the most characteristic features of these stem cells (Figure 9A,D). Bundles of generating axons were demonstrated in direct contact with these stem cells (Figure 9A). Narrow processes of astrocytes were seen among the ECs. The cytoplasm of the astrocytes was electron-dense with fewer microtubules and other organelles and their processes contained granules containing mitochondria (Figure 9C).

**Figure 10:**
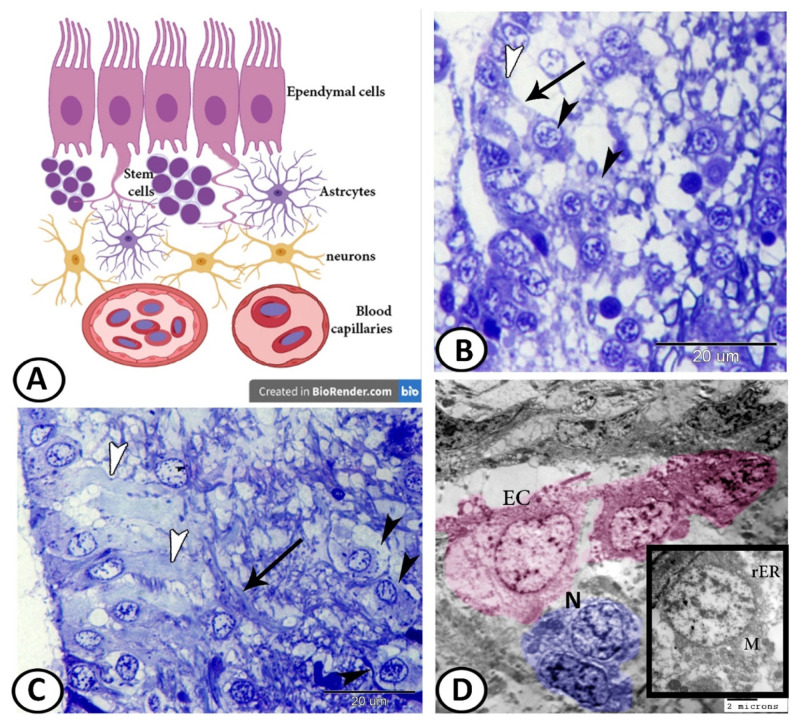
Glia–neuron interaction dynamics. (**A**) Schematic representation of the composition of the ventricular layer of neurogenic niches. (**B**,**C**) Periventricular neurons (black arrowheads) extended their axons (arrow) to the cell processes of ECs (white arrowheads). (**D**) Digital colored TEM image showing the connection between ECs and neurons (N, boxed area), showing mitochondria (M) and rER.

The most interesting finding in the present study was the glia–neuron interaction. The schematic representation illustrates the composition of the ventricular layer of neurogenic niches. It consists of glia, neural stem cells, periventricular neurons, and vascular cells (Figure 10A). The semithin sections show that periventricular neurons with characteristic cytoplasmic Nissl’s granules were observed to neighbor these ECs and extend their axons to the cell processes of the ECs (Figure 10B,C). The results of the TEM confirmed this interaction in which the neurons with cytoplasmic rER and mitochondria established contact with ECs (Figure 10D).

## 4. Discussion

The cellular layer covering the surface of the brain ventricles and spinal canal, known as the ependyma, is important for cerebrospinal fluid (CSF) dynamics [11,35]. The ependymal cells of molly fish have bundles of motile cilia, similar to those present in zebrafish. These cilia beat and contribute to the directional flow of CSF [36]. Ependymal cells constitute the principal neuroglial element in the brains of fish, amphibians, reptiles, and birds [8]. In addition to their role in CSF circulation, ECs are required for the adult neurogenic niche to assemble into its characteristic pinwheel-like architecture [24].

The ependymal cells in the wall of the optic ventricle of molly fish form a continuous layer of ciliated columnar or cuboidal cells. These cells are connected with adjacent cells by desmosomes and gap junctions. The cilia of ependymal cells extend into the optic ventricle, and their long fibrillar processes extend through the full thickness of the tectal laminae and end at the surface of the tectum as a subpial end-foot. Stevenson and Yoon [22] provided a broad description of the ECs in the optic tectum of the goldfish, *Carassius auratus*. They investigated whether ECs had the same description as other teleosts; however, their peripheral processes ramified locally in the tectum region, with the cell body positioned near the tectum ventricular lumen. The ependymal cells in the perciform teleost send a vertical process of fibrils, which runs through the OT and terminates at the pia mater after giving off several short leaf-like processes along its course [37]. The presence of numerous bundles of intermediate filaments or fibrils throughout the cytoplasm of ECs is a main feature of these cells. These bundles of filaments appear homolog to those in astrocytes of the mammalian CNS; therefore, the ECs of teleost are different from mammalian ECs [38].

Several functions have been reported for ependymal cells. Kriebel [39] reported a neuroendocrine function of the ECs lining the ventricular surface. The presence of cytoplasmic intermediate filaments in ECs is a principal feature of teleost ependymal cells. The presence of these filaments allows ECs to function as a supportive barrier between the brain and cerebrospinal fluid. A morphometrical study of EC number is considered an indicator of their function. Decreased number and delayed maturation of ECs may disrupt CSF flow dynamics during early brain development and, consequently, enhance the accumulation of CSF within the brain ventricles, resulting in hydrocephalus [40]. Furthermore, CSF flow dynamics can affect the proliferation of neural stem cells and the migration of neuroblasts towards the olfactory bulb [41].

It is well known that the process of neurogenesis plays a crucial role in the replacement of damaged neurons. Compared with any other studied vertebrate, teleost fish have the most pronounced and extensive adult neurogenesis across the central nervous system [42,43,44,45]. Adult neurogenesis is critical for the numerical matching of neurons in the central nervous system, as well as sensory and motor aspects in fish [45,46]. The ECs showed an expression of myostatin and SOX9 in their cytoplasmic processes. The myostatin precursor was detected in several teleost fish tissues including the brain, where its immunoreactivity was detected in the mesencephalon of sea bream and in the telencephalon of zebrafish, proposing a possible role of myostatin in neuronal growth and development [47]. Myostatin or growth differentiation factor-8 (GDF-8) is a member of the TGF-β superfamily. It is mainly expressed in skeletal muscle where it is implicated in the regulatory process of skeletal muscle growth [48,49]. Recently, myostatin has been reported as a negative regulator of adult neurogenesis in zebrafish [50]. Myostatin-like proteins such as myoglianin have been detected as important inhibitors of neuronal growth and synapse function [51].

The SOX family is essential for stem cell maintenance and embryonic development in humans and zebrafish [52,53,54]. SOX9 is a member of the SOX family, and plays an important role in cell proliferation and cell fate regulation during embryogenesis, where its mutations induce abnormal cellular growth [55,56,57]. Furthermore, SOX9 regulates stem and progenitor cells in adult tissues [58,59], and it is implicated in neural stem cells’ identity [59]. SOX9 is essential for differentiation along the neuronal lineage, both in the adult and embryonic central nervous system [60]. Numerous SOX transcription factors play various roles from the initial differentiation phases to the generation of mature neurons [61,62], and the roles of these transcription factors in the regulation of adult neurogenesis, especially in the hippocampus, have been extensively reported [63,64,65]. Recently, SOX9 transcription factors have been reported as an essential regulator of neuronal and glial differentiation during neural development and adult neurogenesis [66]. Furthermore, SOX9 exerts planned impacts on transcription, neuron production, basal progenitors’ proliferation, and neurogenic cell fate of the embryonic mouse neocortex [67].

ECs showed the expression of IL-1β in their cytoplasmic processes. In fish species, including sharp-tooth fish and air-breathing fish, IL-1β is one of the earliest-expressed pro-inflammatory cytokines, and is secreted by blood monocytes, tissue macrophages, activated endothelial cells, activated T lymphocytes, granulocytes, and other cell types. It mediates the regulation of innate and adaptive immune responses, enabling organisms to respond immediately to infection [68,69,70,71]. Moreover, IL-1β has a critical role in the launch of local and systemic responses to different stimuli via natural killer cells, T and B lymphocytes, and activating macrophages [72,73]. Increased IL-1β production has been reported to be involved in a wide variety of cellular activities, such as cell proliferation, differentiation, and apoptosis [74,75]. Previous studies on Atlantic hagfish, rat, and IL-1β-converting enzyme-deficient mice revealed the aggravative effect of IL-1β on the primary damage induced by central nervous system infection. Furthermore, in in vivo studies, the lack of IL-1β reduced neuronal loss and infarct volumes following ischemic brain damage [76,77,78]. IL-1β has been shown to promote neuronal differentiation through a Wnt5a/RhoA/ROCK/JNK pathway in cortical neural precursor cells [79].

Ependymal cells exhibit strong APG5 cytoplasmic expression and Nrf2 nuclear expression. Autophagy-related gene 5 (APG5 or Atg5) is one of the critical regulators of the autophagy process [80]. It is essential for various processes including lymphocyte development and proliferation, autophagic vesicle formation, and apoptosis [81]. Atg5 plays an essential role in the central nervous system of mice, where its expression increases with cortical development and differentiation. Moreover, the suppression of Atg5 expression inhibits differentiation, promotes cortical neural progenitor cell proliferation, and impairs cortical neuronal cell morphology, confirming the crucial role of Atg5 in the cortical neurogenesis development of embryonic murine brain development [82]. The autophagy–lysosomal pathway has been concluded to regulate adult neural stem cell maintenance, quiescent neural stem cell activation, and newly born neurons’ survival and maturation time [83].

Nrf2 is involved in various processes such as immunopotentiation, antioxidation, and osmoregulation, in addition its role in toxicity and oxidative stress, as described in *Coilia nasus* [84,85]. In addition to its role in modulating the stress response, Nrf2 can control cellular functions, including protein quality, cell proliferation and differentiation, and mitochondrial function in glioma stem cells, mice, and *Drosophila* [86,87,88]. In human embryonic stem cells and also in mice, Nrf2 has been reported to drive critical aspects of embryonic, adult, and induced pluripotent stem cell proliferation, neuronal differentiation, and function [89,90]. Moreover, Nrf2 maintenance is important for proper neural stem/progenitor cell proliferation and differentiation [91,92]. Nrf2 expression induction has been detected to ameliorate the phenotypic defects observed in neural stem cells isolated from the embryonic cortex of frataxin knockin/knockout mice, re-establishing a proper neuronal differentiation program in Friedreich’s ataxia [93].

GFAP is expressed in the ECs of many teleost species, including *Iberian barbel* (ray-finned fish species) [94,95]. GFAP is a major constituent of glial intermediary filaments that form the cytoskeleton of mature astrocytes, and is responsible for maintaining glial cells’ mechanical strength, and supporting neighboring neurons and the blood–brain barrier [96,97]. It is found in astrocytes in the central nervous system, non-myelinating Schwann cells in the peripheral nervous system, and enteric glial cells [98]. In mice, the upregulation of GFAP and vimentin, an intermediate filament protein of astrocytes, is considered the hallmark of astrocyte activation and reactive gliosis following injury, ischemia, or neurodegeneration [99]. GFAP-expressing progenitors have been highlighted as the principal source of constitutive neurogenesis in adult mouse forebrain [100].

The current results show a glia–neuron interaction that may indicate the transport of chemical signals between them. Both neurons and glia interact dynamically to enable axonal signal conduction, synaptic transmission, and information processing, and so are critical for the normal functioning of the nervous system during development and throughout adult life. The signals between neurons and glia include neurotransmitters, ions, cell adhesion molecules, and other signaling molecules released from synaptic and non-synaptic regions of the neuron. Glial cell communication modulates neuronal excitability and synaptic transmission and coordinates the transfer of information across networks of neurons [101]. Interestingly, glial cells of zebrafish possess specific receptors that are required for controlling the expression of genes involved in neuroendocrine regulation [102]. Furthermore, somatostatin is expressed by neurons and glial cells of *A. leptorhynchus* and is probably involved in regulating neurogenesis in response to neural injury for replacing damaged cells [103]. Further studies should be conducted to identify the signaling pathways in which teleostean glia–neurons interact in the teleostean brain during neuronal regeneration.

In the sunfish, some ECs that present in association with blood vessels may play a role in sensing the biochemical composition of both cerebral fluid and blood and transfer this information to neuronal elements [104]. The newly proliferated neurons during the process of neurogenesis should migrate from the source of their creation to the site of injury as a critical step in the subsequent process of the recruitment of new cells for the repair of lesioned tissue. In the adult teleost fish *Apteronotus leptorhynchus*, several lines of evidence show that radial glial fibers are responsible for this process [105].

The most interesting results in this study are the occurrence of stem cells in close positions to ECs. The bundles of generating axons in direct contact with these stem cells indicate the role of ECs in neurogenesis. In this context, Nelson et al. [106] found that the ependymal glia in the transected zebrafish cord form elongated bipolar bridges that span the lesion site and correlate with trans-lesional axon regeneration.

S100 is a multigenic family of Ca^2+^-binding proteins with at least 25 members identified and localized in both the cytoplasm and nucleus of different cells [107,108]. S100 protein is mostly found in non-neuronal cells in the zebrafish central nervous system. It has been investigated in the diencephalon, the optic tectum, and the mesencephalon, as it is identified mainly in the epithelium that lines the brain ventricles, tanycytes, astrocytes, and subependymal radial glial [109]. S-100 protein in the central nervous system has neurotrophic activity and regulates the cytoskeleton stability of cells [110]. Moreover, Grandel et al., in 2006, added that S100 protein continues to be expressed in the glial cells and the subventricular zone of adult zebrafish, which may play an important role in adult neurogenesis [111].

## 5. Conclusions

The findings of this study confirmed the role of ependymal cells (ECs) in the neurogenesis of molly fish. The presence of cytoplasmic intermediate filaments in the ECs allows their function as a supportive barrier between the brain and cerebrospinal fluid. The expressions of myostatin and SOX9 in the ECs suggest their possible role in neuronal growth and development, cell proliferation, and cell fate regulation, in addition to neuronal and glial differentiation during neural development and adult neurogenesis. The immunoreactivity of IL-1β in ECs suggests their critical role in inducing local and systemic responses and promoting neuronal differentiation. The expressions of APG5 and Nrf2 in ECs suggest their role in lymphocyte development and proliferation, autophagic vesicle formation, and immunopotentiation, in addition to proper neural stem/progenitor cell proliferation and differentiation. The immunoreactivity of GFAP in ECs suggests their role in glial cell maintenance. The expression of S100 in the ECs suggests their role in adult neurogenesis. Finally, the presence of stem cells close to ECs and in contact with their generating axons indicates the role of ECs in neurogenesis.

## Data Availability

The data presented in this study are available within the article.
